# Lipid Raft-Mediated Regulation of G-Protein Coupled Receptor Signaling by Ligands which Influence Receptor Dimerization: A Computational Study

**DOI:** 10.1371/journal.pone.0006604

**Published:** 2009-08-11

**Authors:** Mohammad Fallahi-Sichani, Jennifer J. Linderman

**Affiliations:** Department of Chemical Engineering, University of Michigan, Ann Arbor, Michigan, United States of America; German Cancer Research Center, Germany

## Abstract

G-protein coupled receptors (GPCRs) are the largest family of cell surface receptors; they activate heterotrimeric G-proteins in response to ligand stimulation. Although many GPCRs have been shown to form homo- and/or heterodimers on the cell membrane, the purpose of this dimerization is not known. Recent research has shown that receptor dimerization may have a role in organization of receptors on the cell surface. In addition, microdomains on the cell membrane termed lipid rafts have been shown to play a role in GPCR localization. Using a combination of stochastic (Monte Carlo) and deterministic modeling, we propose a novel mechanism for lipid raft partitioning of GPCRs based on reversible dimerization of receptors and then demonstrate that such localization can affect GPCR signaling. Modeling results are consistent with a variety of experimental data indicating that lipid rafts have a role in amplification or attenuation of G-protein signaling. Thus our work suggests a new mechanism by which dimerization-inducing or inhibiting characteristics of ligands can influence GPCR signaling by controlling receptor organization on the cell membrane.

## Introduction

G-protein coupled receptors (GPCRs) play an important role in signal transduction and are encoded by more than 1000 genes in the human genome [1]. It is estimated that more than 50% of pharmaceuticals target GPCRs, leading to initiation or blockage of a signaling cascade that results in a cell response [2]. When stimulated by their specific ligands, GPCRs activate heterotrimeric G-proteins on the cell membrane, inducing GDP-GTP exchange and formation of the GTP-bound G_α_-subunit and release of the G_βγ_-dimer. These G-protein subunits then activate specific secondary effectors, leading to distinct biological functions. The ligand-bound GPCR can be desensitized by a mechanism which involves receptor phosphorylation by G-protein receptor kinase (GRK) and internalization of the receptor followed by either recycling or degradation [3]. Much research is underway to determine the mechanisms by which GPCR signaling is regulated. Here we focus on understanding factors that influence GPCR organization on the cell membrane and how such organization can influence GPCR signaling.

Two mechanisms that affect receptor organization on the cell membrane have been proposed. First, many GPCRs have been shown to form homo- and/or hetero-dimers/oligomers on the cell membrane [1], [4], although the role of such dimer/oligomer formation in GPCR signaling is unclear [5]–[9]. Using a computational model, we recently demonstrated that reversible dimerization of receptors under the diffusion-limited conditions typical of membrane-localized reactions can influence receptor organization [10]. Depending on the values of the dimerization and monomerization rate constants, receptors can be organized in different ways on the two-dimensional surface of the cell. The monomer regime is observed when the rate of receptor monomerization is much greater than the dimerization rate. In the dimer regime, the rate of dimerization is much greater than the monomerization rate. However, when both receptor dimerization and monomerization are fast, “partner switching”, i.e. alternating of bonds between neighboring receptors, occurs quickly, leading to the formation of oligomer-like clusters of receptors on the cell membrane (oligomer regime) (Supplementary Figure S1). Some GPCRs undergo ligand-induced dimerization, while ligand stimulation has either no effect or decreases the level of dimerization in others [4], [11]. Therefore, dimerization-mediated organization of receptors can be affected differently by ligand stimulation.

As a second mechanism of receptor organization, many GPCRs become localized in membrane microdomains, including lipid rafts and caveolae. Lipid rafts are regions of elevated cholesterol and glycosphingolipid content, greater order, and less fluidity within cell membrane [12]. Caveolae are lipid rafts with flask-shaped structures and are distinguished from flat-shaped lipid rafts by the presence of the cholesterol-binding protein caveolin [12]. It has been reported that membrane proteins with at least one transmembrane domain or with a hydrophobic modification are enriched in lipid rafts [13]. Lipid raft-associated proteins diffuse more slowly inside lipid rafts than in non-raft regions, probably due to the tight packing of lipids which leads to a higher local viscous drag on raft proteins [14]. In the simplest model proposed for the role of lipid rafts in GPCR signaling, lipid rafts are viewed as signaling platforms that facilitate interaction of different molecules involved in a specific signaling pathway with a higher density [15]. Compartmentalization of signaling molecules may lead to an increase in activation because of an increased collision frequency between the species [16]. This model may also enhance the specificity of signaling (i.e. reduce crosstalk) when localization of receptors is restricted to a particular class of rafts or when some receptor species are excluded from domains containing other receptor species, although the data on this point are not conclusive [17].

Although dimerization and lipid raft-localization have individually been identified as mechanisms that influence GPCR organization on the cell membrane, some reports have also indicated that localization of membrane proteins in lipid rafts can be affected by their dimerization [18], [19]. This suggests that these two mechanisms of receptor localization must be considered together to understand GPCR localization on the cell surface. We developed a computational model describing GPCR organization on the cell membrane and G-protein activation by ligand-bound receptors. We use our model to answer the following questions: Is GPCR localization in microdomains influenced by dimerization? Why do some GPCRs move into lipid rafts following ligand binding [20]–[22] while others move out of lipid rafts [23], [24] or are not affected [23]? How does GPCR localization in microdomains affect signaling? Why does lipid raft disruption amplify G-protein signaling in some cells but attenuate it in others [24], [25]? Our results suggest that lipid rafts and GPCR dimerization together provide a mechanism by which the cell can regulate G-protein signaling.

## Methods

**Figure 1:**
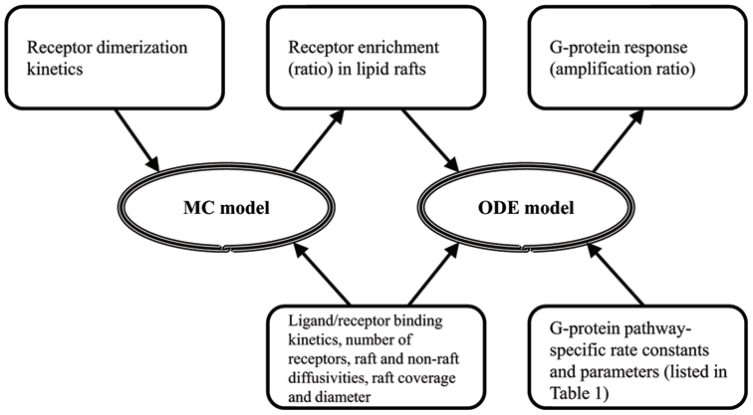
Schematic showing the relationship between the Monte Carlo (MC) model of receptor dimerization and localization and the ordinary differential equation (ODE) model of G-protein signaling. Input parameters are shown by arrows pointing toward the models. Model outputs are shown by arrows pointing away from the models.

To describe GPCR organization on the cell membrane due to dimerization and lipid raft partitioning and the effect of that organization on GPCR signaling, two separate models were used (Figure 1). First, a kinetic Monte Carlo (MC) model was developed to determine the effect of a ligand-induced change in the dimerization status of receptors on localization within low-diffusivity microdomains (lipid rafts) on the cell surface and to estimate the time-scale and level of receptor clustering and declustering. An MC framework allows examination of the roles of stochastic effects and partner switching in receptor organization and quantification of non-homogeneous receptor distributions in membrane microdomains. Second, an ordinary differential equation (ODE) model based on the collision coupling model [26], [27] was developed for studying the effect of receptor localization within lipid rafts on downstream signaling events. Linking this simple model to the MC model allows us to study and analyze G-protein activation while incorporating the effects of receptor organization; continuing to use the MC method for the activation part of the problem adds substantial computational time and complicates the sensitivity analysis without significant benefit. MC and ODE models and their inputs and outputs are linked as depicted in Figure 1.

### Monte Carlo model for receptor dimerization and localization

A two-dimensional lattice was used to represent the cell membrane and cell surface molecules. Simulations were run on a 700 by 700 triangular lattice with periodic boundary conditions and a lattice spacing of 0.5 nm. To simulate lipid rafts, we assigned low diffusivity regions with uniform distribution and defined surface area (2–30% of the cell membrane) as raft regions on the lattice. The diameter of simulated lipid rafts was varied from 20–50 nm in different simulations. The range of parameters for raft coverage and diameter is consistent with a variety of experimental data [13], [14], [28]–[31].

**Figure 2:**
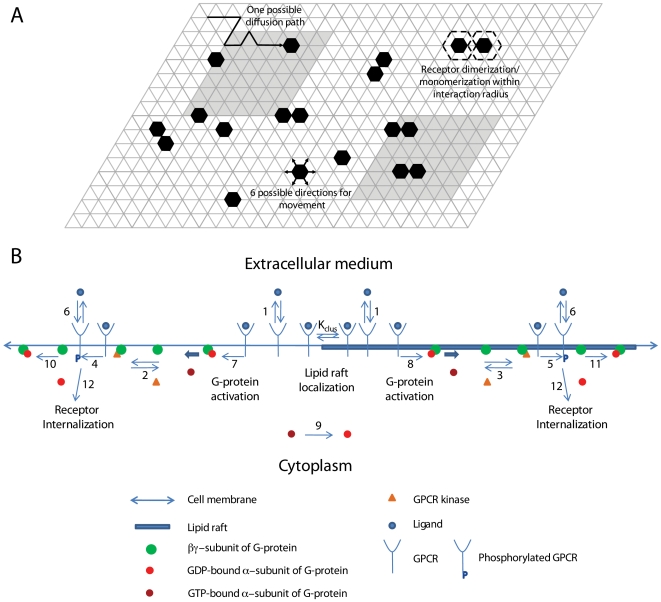
Schematic representation of the structure of (A) the Monte Carlo model of receptor dimerization and localization and a section of the lattice simulating the cell membrane, and (B) the ODE model of G-protein coupled receptor signaling. Black hexagons and gray squares in (A) represent receptors and lipid rafts, respectively. One lattice spacing here is equivalent to 10 real simulation lattice spacings. The ODE model shown in (B) includes ligand binding, ligand-induced lipid raft partitioning of receptors, G-protein activation by receptor-ligand complex, receptor phosphorylation by GPCR kinase, and receptor internalization. Numbers represent model reactions as listed in Table 2. Clustering equilibrium constant *K_clus_* is determined by MC simulations and characterizes receptor enrichment in lipid rafts.

The lattice contained receptor molecules simulated as hexagons with a diameter of 5 nm, the approximate diameter of a single GPCR (Figure 2A). Receptor movement and dimerization was simulated using the algorithm presented by Woolf and Linderman [10]. Briefly, receptors were chosen at random to dimerize with a neighbor, dissociate from a dimerized pair, or diffuse in the plane of the membrane. If the chosen action was a dimerization event, the receptor was first tested to be a monomer. Then, a random neighboring receptor within the “interaction radius” of 5 lattice spacings (2.5 nm) was chosen as a binding partner. If the binding partner was also a monomer, dimerization was allowed with probability *P_dimer_*. If the chosen action was a monomerization event and the receptor was part of a dimer, then monomerization was allowed with probability *P_mono_*. The probabilities of these reactions are derived from the intrinsic reaction rate constants (*k_dimer_*, *k_mono_*). For a diffusion event, receptors moved a single lattice space in a random direction with a probability calculated from the translational diffusion coefficient, *D*, of the protein on the cell membrane. As a result of these diffusion rules, individual receptors move with approximately the same diffusion coefficient regardless of their dimerization state, which is consistent with theoretical findings that show the diffusion is only a weak function of particle radius [32].

In order to study the effect of ligand binding, simulations were run to equilibrium for unligated receptors with specified probabilities of dimerization and monomerization. Ligand at a particular concentration was then added. Receptor/ligand association and dissociation reaction probabilities were calculated based on ligand concentration, receptor/ligand association and dissociation rate constants [33]. Ligand-bound receptors were assumed to participate in dimerization and monomerization reactions with different probabilities from unligated receptors. A more detailed description of the MC simulation procedure is presented in Supplementary Text S1.

To express the level of receptor localization in lipid rafts, we defined the “*enrichment ratio*” as the ratio of the equilibrated number of receptors in lipid rafts over the number of receptors in lipid rafts when receptors are randomly distributed on the cell surface. The enrichment ratio was measured in 1000 simulation runs for each set of parameters and averaged.

Parameter values used in the simulations are listed in Table 1. The intrinsic rate constant for receptor dimerization, *k_dimer_*, describes binding that occurs *after* diffusion has brought two receptors close together. In previous work, we estimated the value of *k_dimer_* to be on the order of 10^5^ s^−1^ by using the GPCR rotational diffusion coefficient of 2.7×10^5^ s^−1^ [32]; a similar value of 10^4^ s^−1^ has been used for dimerization of the epidermal growth factor receptor [34], [35]. Although our MC simulations account for diffusion explicitly by allowing receptors to move among lattice sites, one can also estimate a rate constant *k_+_* for the transport (via diffusion) of one receptor to another (from *k_+_* = 2π*D*/ln(*b*/*s*) where *D* is the translational diffusion coefficient of receptors in the cell membrane, *b* is one-half the mean distance between receptors, and *s* is the encounter radius between two monomeric receptors [26]) of 10^3^–10^5^ s^−1^. *k_+_* is thus likely less than or of the same order as *k_dimer_*, suggesting a diffusion-limited or partially diffusion-controlled reaction in the membrane [26] for which MC simulations are well-suited. Values for the intrinsic monomerization rate constant (*k_mono_*) similar to *k_dimer_* are used, consistent with other work [34]. Diffusivity (*D*) was assumed to be in the range of 10^−10^–10^−9^ cm^2^/s for non-raft regions and 10^−12^–10^−11^ cm^2^/s for low-diffusivity raft regions on the cell surface. These values are consistent with the lower and upper limits of cell membrane diffusivity for membrane receptors [26], [36], [37]. The simulation time step was chosen such that the probability of the most likely event was ∼20%. Simulations were run with 100–1000 particles corresponding to a surface coverage of 1.8–18%. This range of receptor density is consistent with the density of GPCRs that form homo- and hetero-dimers on the membrane of different cell lines used in G-protein signaling experiments [38].

**Table 1:** Model parameter values.

**MC model**			
**Parameter**	**Definition**	**Value**	**Reference**
*k_dimer_* (s^−1^)*	Receptor dimerization rate constant	10^3^–10^7^	[10], [34]
*k_mono_* (s^−1^)	Receptor monomerization rate constant	10^3^–10^7^	[10], [34]
*k_f_* (M^−1^s^−1^)	Ligand/receptor association rate constant	10^8^	[26], [52]
*k_r_* (s^−1^)	Ligand/receptor dissociation rate constant	1	[52]
*D_raft_* (cm^2^/s)	Membrane diffusivity in the raft region	10^−12^–10^−11^	[14], [26], [36], [37]
*D_non-raft_* (cm^2^/s)	Membrane diffusivity in the non-raft region	10^−10^–10^−9^	[14], [26], [36], [37]
*R* (%)	Lipid raft coverage	2–30	[13], [14], [28], [30], [31]
*d* (nm)	Lipid raft diameter	20–50	[13], [14], [28], [30], [31]
**ODE model**			
**Parameter**	**Definition**	**Value** †	**Reference**
*k_f_* (M^−1^s^−1^)	Ligand/receptor association rate constant	10^7^–10^8^ (10^8^)	[26], [52]
*k_r_* (s^−1^)	Ligand/receptor dissociation rate constant	0.1–1 (1)	[52]
*k_f_'* (M^−1^s^−1^)	Ligand/phosphorylated receptor association rate constant	10^6^–10^9^ (10^8^)	[52]
*k_r_'* (s^−1^)	Ligand/phosphorylated receptor dissociation rate constant	0.001–0.005 (0.002)	[52]
*k_on_* (M^−1^s^−1^)	Receptor/kinase association rate constant	10^9^–10^11^ (10^11^)	[52]
*k_off_* (s^−1^)	Receptor/kinase dissociation rate constant	10–100 (25)	[52]
*k_int_* (s^−1^)	Receptor internalization rate constant	10^−4^–10^−1^ (10^−2^)	[52], [74]
*k_rec_* (M^−1^s^−1^)	G-protein recombination rate constant	6×10^9^–6×10^11^ (1.6×10^10^)	[52]
*k_hyd_* (s^−1^)	GTP hydrolysis rate constant	0.02–30	[70], [75]–[77]
*R_tot_* (#/cell)	Total number of cell surface receptors	5×10^4^–5×10^5^ (2.5×10^5^)	[52]
*G_tot_* (#/cell)	Total number of G-proteins	10^4^–10^5^ (7.5×10^4^)	[52]
[*L*]*/K_d_*	Scaled ligand concentration	0.1–10	
*RK_tot_* (M)	Total concentration of GPCR kinase	1.5×10^−9^–3×10^−9^ (3×10^−9^)	[52]
*r*	Relative G-protein density	0.02–0.8	
*D_non-raft_* (cm^2^/s)	Membrane diffusivity in the non-raft region	10^−10^–10^−9^ (10^−10^)	
*k_c_, k_c_'* (M^−1^s^−1^)	G-protein activation rate constant	Computed from Equation (1)	
*K_clus_*	Clustering equilibrium constant	Found from MC simulation	
*k_p_, k_p_'* (M^−1^s^−1^)	Receptor phosphorylation rate constant	Computed similarly to *k_c_* and *k_c_'*	
*D_non-raft_/D_raft_*	Ratio of non-raft diffusivity to lipid raft diffusivity	10	

*k_dimer_ is an intrinsic rate constant, meaning that it describes the rate at which binding takes place after diffusion has brought the proteins within reaction range.

† Ranges of parameters shown for the first 15 parameters (all independent) are used for sensitivity analysis. Values in parentheses are used to generate model results shown in Figures 6–8.

### ODE Model for GPCR signaling

Our model for GPCR signaling incorporates ligand binding, lipid raft partitioning of receptors due to ligand binding (i.e. the enrichment ratio as determined by the MC model), G-protein activation by receptor-ligand complexes (both within and outside of lipid rafts), receptor phosphorylation by GPCR kinase, and receptor internalization as shown in Figure 2B. G-proteins were assumed to be highly enriched in membrane microdomains (lipid rafts and caveolae) and did not translocate into/out of them during the time course of simulation. This assumption is based on a variety of experiments showing (more than 10-fold) enrichment of G-proteins in membrane microdomains and preferential interaction of G-proteins with microdomain-specific proteins such as caveolin [13], [39]–[44]. Phosphorylated receptors were considered to be desensitized. The reactions and equations to describe the ODE model are listed in Table 2. Definitions and values of parameters are given in Table 1. The ligand concentration, [*L*], was assumed to remain constant (no depletion). Equations were solved numerically using MATLAB 7.5 (The MathWorks, Natick, MA).

**Table 2:** Description of the reaction species, reactions and equations of the ODE model.

**Reaction species**			
*L*	Ligand	*G_clus_*	Trimeric G-protein in the raft region
*R*	G-protein coupled receptor	*(βγ)_clus_*	βγ-subunit of G-protein in the raft region
*LR*	Ligand/receptor complex	*RK*	GPCR kinase
*LR_scat_*	Ligand/receptor complex in the non-raft region	*LR-P*	Phosphorylated ligand-bound receptor
*LR_clus_*	Ligand/receptor complex in the raft region	*R-P*	Phosphorylated receptor
*G_scat_*	Trimeric G-protein in the non-raft region	*LR_i_*	Internalized ligand-bound receptor
*α-GTP*	GTP-bound (active) α-subunit of G-protein	*α-GDP*	GDP-bound α-subunit of G-protein
*(βγ)_scat_*	βγ-subunit of G-protein in the non-raft region		
**ODE model reactions and flux expressions**			
1	L+R ↔ LR	7	[LR_scat_]∶G_scat_ → α−GTP+βγ_scat_
	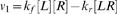		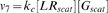
2	βγ_scat_+RK ↔ βγ−RK_scat_	8	[LR_clus_]∶G_clus_ → α−GTP+βγ_clus_
	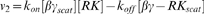		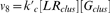
3	βγ_clus_+RK ↔ βγ−RK_clus_	9	α−GTP → α-GDP
	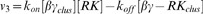		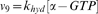
4	[βγ−RK_scat_]∶LR_scat_ → LR−P_scat_	10	α−GDP+βγ_scat_ → G_scat_
	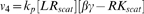		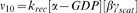
5	[βγ−RK_clus_]∶LR_clus_ → LR−P_clus_	11	α−GDP+βγ_clus_ → G_clus_
	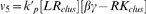		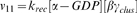
6	L+R−P ↔ LR−P	12	LR−P → LR_i_
	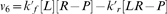		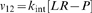
**ODE model equations**			
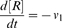		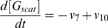	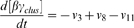
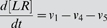		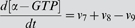	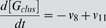
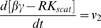		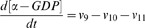	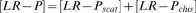
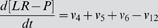		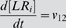	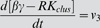
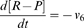		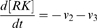	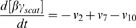
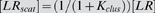		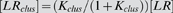	

G-protein activation and receptor phosphorylation were assumed to be diffusion-limited reactions in the membrane [45]. The rate constants for diffusion-limited activation of G-protein by receptor/ligand complex were estimated separately for the non-raft and raft regions using [26]:
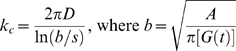
(1)where *D* is the diffusion coefficient, *b* is half of the mean separation distance between reactants, *s* is the encounter radius, *A* is the surface area of the raft or non-raft region, and [*G(t)*] is the time-dependent inactive G-protein concentration ([*G_clus_*(*t*)] in the raft and [*G_scat_*(*t*)] in the non-raft region as defined in Table 2). This estimation is based on the assumption that reactants are well-mixed on the surface of the raft or non-raft regions, while they have different concentrations in each region. If the reactants are locally enriched or depleted in one area, the well-mixed assumption may not be realistic and can be more accurately determined by MC simulations [16], [46]. However, these estimations are similar for the situations described here. The rate constant for receptor phosphorylation was similarly estimated for the raft and non-raft regions. We assumed the total surface area of a cell and the encounter radius, *s*, to be 1000 µm^2^ and 10 nm respectively. The surface area of the raft and non-raft regions was determined from the raft diameter and the total raft coverage.

The distribution of G-proteins may influence the way in which lipid rafts contribute to GPCR signaling. In order to express the pattern of G-protein distribution on the cell membrane (which is not varied over the time course of one simulation), relative G-protein density (*r*) was defined as the ratio of number of (active and inactive) G-proteins in lipid rafts over the total number of G-proteins in the membrane (*G_clus_*|*_t_*_ = 0_ = *r*×*G_tot_* and *G_scat_*|*_t_*_ = 0_ = (1−*r*)×*G_tot_*). Thus, *r* determines the available amount of G-protein for signaling in the raft and non-raft regions. Further, to understand how receptor localization within lipid rafts influences G-protein signaling, the maximum level of G-protein activation was measured as the response in different simulations. This level was used to produce dose-response curves. We defined the “*amplification ratio*” as the ratio of the maximum level of G-protein activation in the presence of lipid rafts to that in the absence of lipid rafts. Amplification ratio values of more than one show that the presence of lipid rafts leads to signal amplification. Amplification ratio values of less than one show that G-protein signal is attenuated by lipid rafts.

### Sensitivity Analysis

Parameter sensitivity of the MC model output (enrichment ratio) was explored by changing input parameters within the ranges specified in Table 1. To identify parameters that significantly influence the outcome of lipid raft-mediated G-protein signaling (signal amplification or attenuation, as calculated by the ODE model), we used Latin hypercube sampling (LHS) [47] to sample values of 15 parameters from the ranges listed in Table 1. A logarithmic distribution was used for ligand concentration and uniform distributions were used for other parameters. Simulations sampled each parameter 1000 times, producing 1000 solutions to the model equations. To determine the correlation between parameter values and the model outcome, amplification ratio, partial rank correlation coefficient (PRCC) values were calculated. PRCC values vary between -1 (perfect negative correlation) and 1 (perfect positive correlation) and can be differentiated based on *p*-values derived from Student's *t* test. Fisher's *z* test was performed to assess if two PRCC values are significantly different from each other [48].

## Results and Discussion

### Receptor localization within lipid rafts can be controlled by dimerization

**Figure 3:**
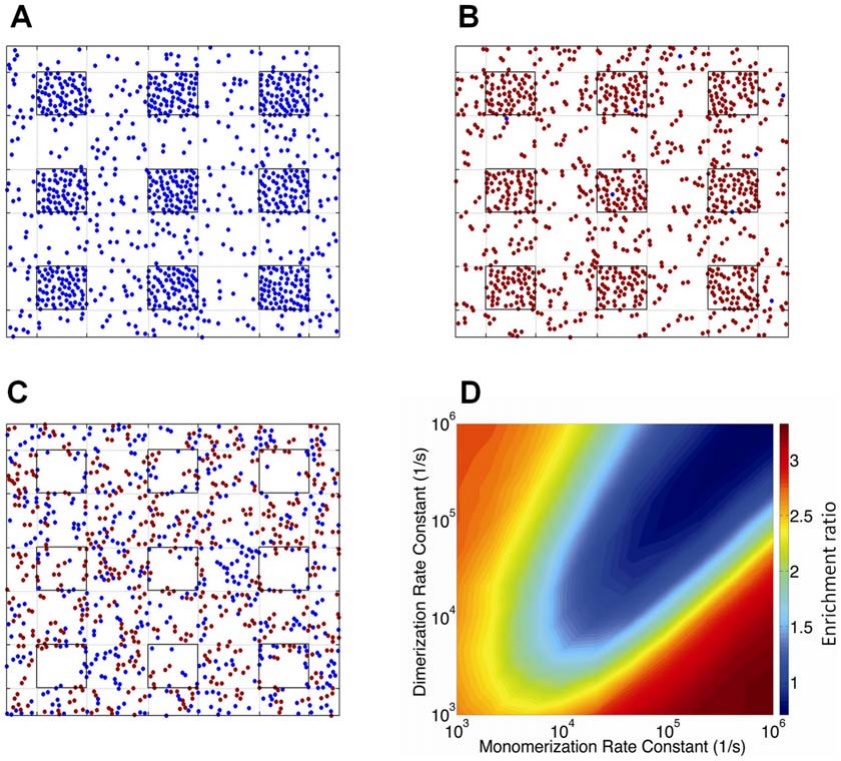
Model-generated organization (snapshots) of receptors diffusing on a cell membrane with low-diffusivity microdomains (lipid rafts): (A) monomer regime (*k_mono_* = 10^6^ s^−1^, *k_dimer_* = 10^3^ s^−1^), (B) dimer regime (*k_mono_* = 10^3^ s^−1^, *k_dimer_* = 10^6^ s^−1^), and (C) oligomer regime (*k_mono_* = 10^6^ s^−1^, *k_dimer_* = 10^6^ s^−1^). Lipid raft regions are shown as nine small squares. Monomers and dimers are shown with blue and brown dots respectively. (D) The predicted enrichment ratio varies with *k_mono_* and *k_dimer_*. Diffusion coefficients in lipid raft and non-raft regions for A-D were set to 10^−12^ and 10^−10^ cm^2^/s respectively. Simulation results with other values of diffusivity are shown in Supplementary Figure S3. Simulations were run to equilibrium with receptor density of 18%. In this set of simulations, rafts make up 20% of the simulated membrane and the raft diameter is 50 nm.

To understand whether localization of membrane receptors into low-diffusivity microdomains (lipid rafts) on the cell surface is influenced by receptor dimerization, MC simulations were run for different values of the rate constants for receptor dimerization and monomerization, assuming diffusion of particles is reduced in specified regions (lipid rafts) on the lattice. When simulations were run with a small value of the ratio *k_dimer_*/*k_mono_*, the monomer regime was observed, driving the equilibrium toward translocation of receptors into lipid rafts (Figure 3A). This is consistent with a recent model describing motion of monomeric particles on a cell membrane including low-diffusivity lipid rafts [49], [50]. When the ratio *k_dimer_*/*k_mono_* was large, the dimer regime was observed, and receptors still translocated into lipid rafts (Figure 3B). Particles (either dimeric or monomeric receptors) in the dimer or monomer regimes move almost independently on the surface. Existence of low-diffusivity regions on such a surface can limit particle movements, leading to crowding of receptors in these regions.

Between the two extremes (very large and very small values of the ratio *k_dimer_*/*k_mono_*), receptors on the cell surface are ordered in oligomer-like structures via the partner switching mechanism (recall Supplementary Figure S1). Interestingly, the equilibrium lipid raft concentration of receptors in the oligomer regime is lower than in the monomer and dimer regimes (Figure 3C). In the oligomer regime, movements of particles can be affected by interactions which are due to the fast dimerization and monomerization reactions. This leads to the formation of oligomer-like structures of receptors which can move together. To test this, an average interaction time was defined as the time two randomly selected interacting particles (either dimer or monomer) spent at a distance of not longer than the previously defined interaction radius from each other, normalized to the time that all particles move on average one lattice spacing. The average interaction time was measured for different regimes in different simulations, and for the oligomer regime was shown to be up to 4 times greater than the monomer or dimer regimes, depending on diffusion conditions, receptor concentration and the rates of receptor dimerization and monomerization (Supplementary Figure S2). This suggests that receptors in a cluster in the oligomer regime move together on the cell surface. Larger clusters are formed in more diffusion-limited conditions [10]. When the cell surface is composed of two distinct regions, one with lower diffusivity (raft region) and one with higher diffusivity (non-raft region), small receptor clusters formed in the high-diffusivity region may enter the low-diffusivity region. Similarly, larger receptor clusters formed in the low-diffusivity region may enter the high-diffusivity region. At equilibrium, this leads to a lower receptor concentration in the low-diffusivity region (Figure 3C). Thus dimerization status influences the localization of GPCRs in lipid rafts.

### Enrichment of receptors in lipid rafts depends on receptor dimerization kinetics and membrane diffusivity

Receptor enrichment in lipid rafts (as defined in Methods) was chosen as a simple metric to study the combined effects of low-diffusivity lipid rafts and receptor dimerization on organization of receptors on the cell membrane. Figure 3D shows the simulation results for variation of the enrichment ratio with dimerization and monomerization rate constants given different values of diffusivity in lipid rafts and non-raft regions of the cell membrane. The minimum enrichment ratio is observed when dimerization and monomerization rates are both large compared to the rate of diffusion and have the similar order of magnitudes (oligomer regime, Figure 3C). However, when *k_dimer_*≫*k_mono_* (dimer regime, Figure 3B) or *k_dimer_* ≪*k_mono_* (monomer regime, Figure 3A), receptors instead tend to translocate into lipid rafts. Significantly, we predict that the enrichment ratio is a ligand-dependent parameter based on experimental data showing that dimerization status of many GPCRs can be altered by the presence or absence of ligands [4], [11].

Diffusivity of receptors in the raft and non-raft regions also influences the organization of receptors. Comparison of Figure 3D with Supplementary Figure S3 shows that as the difference between diffusivities of lipid rafts and non-raft regions is increased, the difference between the maximum and minimum values of enrichment ratio increases. Furthermore, lower values of diffusivity for raft and non-raft regions favor the raft-leaving of receptors with lower values of dimerization and monomerization rate constants. For example, using a value of 10^−10^ cm^2^/s for *D_non-raft_* is sufficient for observing receptor partitioning phenomena in the oligomer regime with an order of magnitude smaller values of *k_dimer_* and *k_mono_* (<10^4^ s^−1^) compared with the case of *D_non-raft_* = 10^−9^ cm^2^/s (compare Figure 3D with Supplementary Figure S3). Thus partitioning of GPCRs into lipid rafts depends on both dimerization and diffusion rates.

### Enrichment of receptors in lipid rafts depends weakly on raft diameter, modestly on total raft area and strongly on the number of receptors

**Figure 4:**
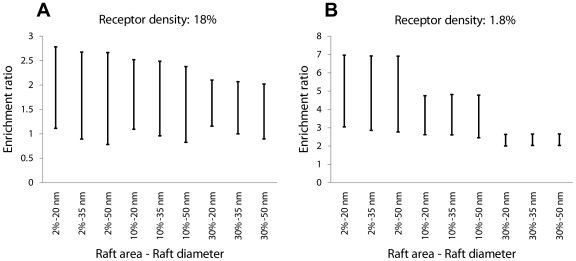
Predicted variation in the enrichment ratio with *k_mono_* and *k_dimer_* as a function of the total area (as a percentage of the cell membrane area) and diameter of lipid rafts. Results are shown for receptor densities of (A) 18% and (B) 1.8% of the cell surface area. For each pair of raft diameter and raft coverage, *k_mono_* and *k_dimer_* are varied from 10^4^ to 10^7^ s^−1^ so as to include monomer, dimer and oligomer regimes. Diffusion coefficients in lipid raft and non-raft regions are 10^−11^ cm^2^/s and 10^−10^ cm^2^/s respectively.

Cell-specific parameters such as raft diameter, raft area, and receptor number may also influence receptor organization. We next examined the effect of the size of a single raft and total lipid raft area on the membrane organization of receptors. Figure 4 indicates simulation results for the range of dimerization-mediated enrichment of receptors in lipid rafts for two distinct numbers of receptors on the cell membrane. Enrichment of receptors in lipid rafts depends weakly on raft diameter. However, total raft area significantly influences the range of dimerization-mediated receptor enrichment in lipid rafts. Figure 4 shows that increasing the area of cell membrane covered by lipid rafts limits the range of variation of enrichment ratio with dimerization and monomerization rate constants. Dependency of receptor enrichment on lipid raft characteristics has a clear biological relevance. Partitioning of receptors with small non-caveolae rafts with a small cell surface coverage and their localization with larger caveolae that occupy 4–35% of the cell membrane area are expected to have different consequences [51].

Decreasing the total number of receptors on the cell membrane leads to a higher enrichment ratio in low-diffusivity raft regions (Figure 4). With fewer receptors, interactions between particles are reduced, leading to the behavior seen for independent particles, i.e. translocation into low diffusivity regions. Thus cell-specific parameters (raft diameter and number, receptor number, diffusivities) as well as ligand-dependent parameters (ability of GPCR to dimerize when bound, or not, by ligand) control GPCR organization or partitioning into lipid raft regions.

### Ligand-induced dimerization-mediated partitioning of receptors with lipid rafts is rapid

**Figure 5:**
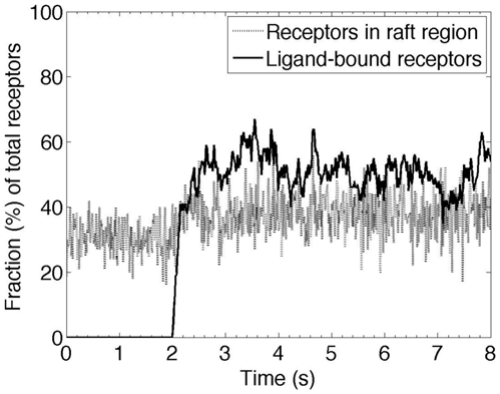
Rapid ligand-induced localization of receptors within lipid rafts due to a ligand-induced change in dimerization kinetics of receptors. Simulation was initiated with randomly distributed receptors on the membrane. Receptors were allowed to equilibrate between monomer and dimer states in the absence of ligand with *k_mono_* = 10^6^ s^−1^ and *k_dimer_* = 10^6^ s^−1^. Ligand with concentration [*L*] = *K_d_* = *k_r_*/*k_f_* was then added and simulations continued until a steady state was reached; *k_f_* = 10^8^ M^−1^s^−1^ and *k_r_* = 1 s^−1^. Ligand-bound receptors were assumed to have the same monomerization rate constant as the unligated state but *k_dimer_* was decreased to 10^4^ s^−1^. Simulations were run on a membrane including lipid rafts with total area of 10% and diameter of 20 nm. Diffusion coefficients in lipid raft and non-raft regions were 10^−11^ cm^2^/s and 10^−10^ cm^2^/s respectively.

Dimerization-mediated partitioning of GPCRs into lipid rafts will only be relevant to determining G-protein activation if it occurs quickly. The simulations presented thus far have examined only steady state behavior. To determine how rapidly the effect of ligand-induced changes in dimerization kinetics can result in receptor re-organization on the membrane, MC simulations were run for different concentrations of ligand, receptor/ligand binding kinetics, and dimerization kinetics. Simulation results indicated that ligand-induced receptor re-organization is rapid compared with ligand binding. One scenario is shown in Figure 5; here rate constants were set such that ligand binding reduced the rate of receptor dimerization and led to an increase in the number of receptors (due to a shift from the oligomer regime to the monomer regime) in lipid rafts. For the simulation shown, lipid raft partitioning of receptors due to ligand binding is rapid, occurring approximately 0.1 s following ligand binding. Thus receptor re-organization occurs quickly enough to be relevant to signaling.

Because receptor re-organization is rapid compared with ligand binding, in later modeling (below) we simply assume that receptor enrichment in lipid rafts following ligand binding can be predicted from the MC model based on the equilibrated concentration of receptors in the raft region. The alternative approach of fitting the MC simulation results to receptor clustering and declustering reactions and using the estimated rate constants for receptor clustering and declustering in the ODE model gave nearly identical results (data not shown).

### G-protein signaling may be amplified or attenuated by lipid rafts

In order to study the effect lipid rafts have on G-protein signaling, predicted values for receptor enrichment in lipid rafts (determined in the MC model) were used in an ODE model for G-protein signaling (Figures 1, 2). Sensitivity analysis (see Methods) was used to identify parameters that quantitatively and qualitatively affect the level of G-protein signaling resulting from GPCR binding.

**Figure 6:**
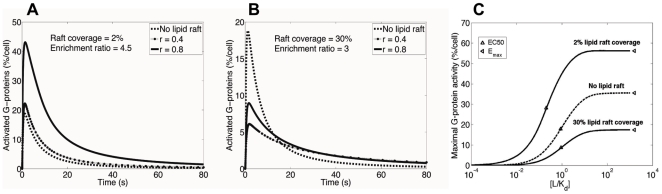
Simulation results for G-protein activation as a function of time at (A) 2% and (B) 30% raft coverage for changing values of *r*, the relative G-protein density in lipid rafts, (C) Simulation results for maximal G-protein activity as a function of scaled ligand concentration for different values of raft coverage. Lines marked “no lipid raft” show the predicted level of G-protein activation in the absence of lipid rafts assuming random distribution of G-proteins on the cell membrane. Although G-protein signaling is attenuated at 30% raft coverage, the rate of termination of the response is smaller compared with no lipid raft condition. This occurs due to reduced rate of (G_βγ_-dependent) diffusion-limited phosphorylation (and thus desensitization) of receptors. Magnitudes of the commonly measured pharmacological parameters maximal effect, E_max_, and half maximal effective concentration, EC_50_, are marked in (C). Parameter values are as listed in Table 1 with *k_hyd_* = 10 s^−1^ and [*L*] = *K_d_* in (A) and (B).

Two regimes of signaling behavior were identified in the model. In the first regime, lipid rafts enhance G-protein signaling. The G-protein activation as a function of time for a specific value of receptor enrichment (enrichment ratio = 4.5) and several different values of the G-protein density in lipid rafts that are 35 nm in diameter and cover 2% of the plasma membrane is shown in Figure 6A. The time course and the level of predicted response are qualitatively consistent with a variety of G-protein signaling experimental and modeling data such as [52], [53], suggesting that our model captures the essential features of GPCR signaling. When receptors are clustered into these relatively small and sparsely distributed lipid rafts following ligand stimulation, increasing the relative density *r* of G-protein in lipid rafts leads to an increase in the maximum level of response. The highest value of relative G-protein density shown (*r* = 0.8) is consistent with experimental data on G-protein enrichment in lipid rafts [13]. Note that the diffusion of GPCRs was assumed to be slower in lipid rafts compared to the non-raft region. This has a negative effect on the rate of diffusion-limited G-protein activation by activated ligand-bound receptors in lipid rafts. However, high levels of G-protein enrichment and receptor localization in lipid rafts provide a high density of reactants which can result in signal amplification compared with G-protein signaling without lipid rafts.

In the second regime, lipid rafts attenuate G-protein signaling. This occurs at larger values of lipid raft coverage (Figure 6B). Scatter plots for the effect of G-protein enrichment in lipid rafts (relative G-protein density) on the model outcome, signal amplification ratio, at three levels of lipid raft coverage are indicated in Supplementary Figure S4. These plots (and also Figures 6A, B) show that the amplification ratio significantly depends on lipid raft coverage. As described earlier, the diameter of a single raft and total lipid raft coverage can significantly affect dimerization-mediated localization of receptors in lipid rafts. Further, lipid raft coverage influences lipid raft-mediated G-protein signaling by controlling the density of membrane signaling molecules in the raft region. As such, signal attenuation (amplification ratio <1) is the general consequence of the presence of lipid rafts at higher levels of coverage (10 or 30%), where the negative effect of low diffusivity in lipid raft dominates G-protein signaling. However, 2% lipid raft coverage provides a sufficient level of receptor and G-protein enrichment in lipid rafts to amplify G-protein signaling (amplification ratio >1). In addition, G-protein enrichment in lipid rafts is positively correlated with amplification ratio and this correlation is significantly stronger for 2% lipid raft coverage than 10 or 30% coverage (via Fisher's *z* test).

Experimental studies on the role of lipid rafts in GPCR signal transduction are done indirectly by examining the effect of disruption of lipid rafts by cholesterol depletion using agents such as methyl-β-cyclodextrin on GPCR signaling. Cholesterol depletion generally impairs G-protein mediated signaling, indicating that the presence of lipid rafts enhances G-protein signaling [22], [54], [55]. This effect can be explained by the first regime of signaling behavior in our model. However, in some systems disruption of lipid rafts has a positive effect on GPCR signaling, indicating that G-protein signaling may also be diminished by lipid rafts as explained by the second regime [24], [25], [56], [57]. The effect of lipid raft disruption experiments on the G-protein response can be tracked by comparing the dose-response curves displayed in Figure 6C (and also curves in Figure 6A, B) in the case of no lipid rafts with those in the presence of lipid rafts.

Although the general effect of lipid raft disruption on G-protein signaling (change in the level of response) has been assessed via the experiments referenced above, the significance of different physical processes represented in our model in lipid raft-mediated G-protein signaling has not been studied. There are other parameters (besides raft coverage) that also affect amplification ratio. Table 3 indicates the rank order of PRCC values for model parameters at three levels of lipid raft coverage. Amplification ratio was shown to be influenced by a variety of cell-specific parameters that most importantly include G-protein enrichment in lipid rafts (relative G-protein density, *r*), GTP hydrolysis rate constant (*k_hyd_*), diffusivity (*D_non-raft_*), total number of cell surface receptors (*R_tot_*) and G-proteins (*G_tot_*), as well as ligand concentration (*L*). Although the parameters that were highly correlated did not differ much between 10% and 30% lipid raft coverage, a significantly distinct pattern of correlation was observed for 2% lipid raft coverage. Ligand concentration, total number of cell surface receptors and diffusivity in non-raft region are parameters that negatively correlate with amplification ratio in G-protein signaling at 2% lipid raft coverage but positively correlate with amplification ratio at higher levels of lipid raft coverage. On the other hand, decreasing the GTP hydrolysis rate constant (*k_hyd_*) reduces amplification ratio when lipid rafts cover 2% of the cell membrane but increases amplification ratio when lipid rafts cover 10 or 30% of the cell membrane. Indeed, the four parameters mentioned above all act to strengthen the effect of the presence of lipid rafts on G-protein signaling, so that greater *k_hyd_*, for example, induces greater amplification when lipid rafts lead to signal amplification (2% lipid raft coverage), but intensifies signal attenuation when lipid rafts attenuate G-protein signaling (10 or 30% lipid raft coverage).

**Table 3:** Parameters significantly correlated with amplification ratio.

2% lipid raft coverage		10% lipid raft coverage		30% lipid raft coverage	
*r*	0.89	*k_hyd_*	−0.65	*k_hyd_*	−0.76
*G_tot_*	0.59	*D_non-raft_*	0.63	*D_non-raft_*	0.75
[*L*]	−0.20	*r*	0.48	*r*	0.63
*k_hyd_*	0.20	*R_tot_*	0.46	*R_tot_*	0.63
*k_on_*	0.20	*G_tot_*	0.40	[*L*]	0.48
*R_tot_*	−0.19	[*L*]	0.37	*k_r_*	−0.34
*k_rec_*	−0.16	*k_on_*	0.29	*G_tot_*	0.31
*D_non-raft_*	−0.12	*k_off_*	−0.22	*k_on_*	0.19
		*k_r_*	−0.17		
		*k_rec_*	−0.12		

PRCC values of model parameters are listed in rank order of correlation. Parameters with significant PRCC values (*p*<0.001) are listed.

The correlation of GTP hydrolysis rate constant *k_hyd_* with amplification ratio suggests a role for RGS proteins in lipid raft-mediated G-protein signaling. RGS proteins enhance GTP hydrolysis, thus reducing the concentration of activated G-protein. However, such enhancement exerts differential effects in the raft and non-raft regions of the membrane. Greater enrichment of reactants in lipid rafts at 2% coverage leads to more rapid re-activation of G-protein following GTP hydrolysis compared with that in the non-raft region or when reactants are randomly distributed on the membrane due to lipid raft disruption. In other words, receptor and G-protein enrichment in lipid rafts but not in the non-raft region compensates for G-protein deactivation by RGS, leading to a larger signal amplification ratio overall. However, at 10 or 30% lipid raft coverage, re-activation of G-proteins in the raft region following GTP hydrolysis is not sufficiently rapid (and is even slower than the non-raft region) to compensate for G-protein deactivation in the presence of RGS. This explains the negative correlation of *k_hyd_* with amplification ratio at 10 and 30% lipid raft coverage (see Table 3).

### Dimerization can act as a tool for regulating GPCR signaling

**Figure 7 pone-0006604-g007:**
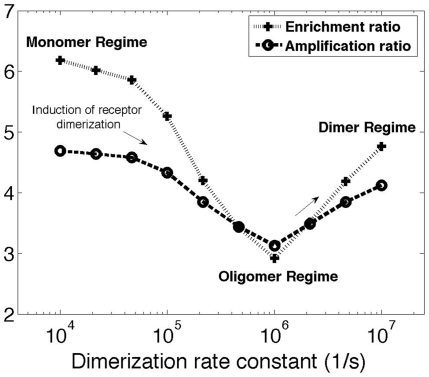
Regulation of the G-protein response by dimerization-mediated enrichment of receptors in lipid rafts. Combination of the results of the Monte Carlo and the ordinary differential equation models are indicated for the effect of ligand-induced receptor dimerization on lipid raft-mediated G-protein signaling at 2% raft coverage. Monomerization rate constant was maintained constant (*k_mono_* = 6.7×10^5^ s^−1^). Results are shown for ligand concentration: [*L*] = 0.1*K_d_*, *k_hyd_* = 30 s^−1^. Membrane diffusivities in the raft and non-raft regions are the same as Figure 6A. Other parameter values are as listed in Table 1. The qualitative aspects of this plot are similar for large values of lipid raft coverage, except that the amplification ratio values are less than one.

Taken together, the results presented above demonstrate that dimerization may act to enrich or deplete the number of GPCRs in lipid rafts, and that lipid rafts may serve to either amplify or attenuate G-protein signaling. The effect of ligand-induced receptor dimerization on G-protein signaling is summarized in Figure 7, which includes results from both the MC and the ODE model. The maximum level of G-protein activation (as described by the amplification ratio) depends on receptor enrichment in lipid rafts (enrichment ratio) and receptor enrichment itself can be regulated by ligand-dependent receptor dimerization kinetics. The greatest receptor enrichment in lipid rafts is observed in the monomer regime (when *k_dimer_*≪*k_mono_*). Increasing the dimerization rate constant without changing the monomerization rate constant results in a shift from the monomer regime to the oligomer regime. This moves receptors out of lipid rafts, leading to a lower level of response. However, a further increase in the dimerization rate constant to values larger than the monomerization rate constant (*k_dimer_*≫*k_mono_*) shifts receptors to the dimer regime, leading to a greater enrichment in lipid rafts and thus a higher level of response. This pattern is qualitatively similar for G-protein enriched lipid raft-mediated signaling at small and large lipid raft coverage (data not shown). These results indicate that receptor clustering could be used as a tool for regulating GPCR signaling, particularly in the context of G-protein distribution which can also be regulated, for example via priming [58].

### Modeling results are consistent with unexplained experimental data on receptor distribution and lipid raft-mediated GPCR signaling

We now compare our modeling results with experimental data, beginning first with the predictions of the MC model. As summarized most clearly in Figure 7, in different GPCR systems ligand-induced receptor dimerization can exert opposite effects on the level of receptor enrichment in lipid rafts, depending in large part on the regime (monomer, oligomer and dimer) of unligated receptors. Our finding that a ligand-induced change in dimerization kinetics can cause translocation of receptors into or out of lipid rafts is consistent with unexplained experimental data on GPCRs. For example, δ-opioid receptors have been shown to exist as dimers on the membrane of CHO cells [59], and a majority (approximately 70%) of the receptors on CHO cell membranes are located in lipid rafts [24]. This is consistent with our model results showing that receptors in the dimer regime translocate into lipid rafts. Further, the level of dimerization is agonist-dependent; increasing concentrations of etorphine reduce the level of receptor dimerization [59]. Etorphine treatment has been shown to move more than 20% of raft-associated receptors out of lipid rafts [24], consistent with our modeling results for shifting from the dimer regime to the oligomer regime. In contrast to etorphine, naloxone, an inverse agonist for δ-opioid receptors, does not affect receptor dimerization [59] and thus our model does not indicate any significant changes in distribution of receptors relative to lipid rafts, consistent with experimental observations [24], [59].

As a second example, although ligand binding has been found to induce dimerization of both β2-adrenergic receptors on Sf9 cells and bradykinin B2 receptors on PC-12 cells [60], [61], it has distinct effects on receptor localization with lipid rafts. While ligand binding causes translocation of the β2-adrenergic receptors out of lipid rafts, it leads to bradykinin B2 receptor clustering in lipid rafts [12]. Our modeling indicates that unligated β2-adrenergic receptors on Sf9 cells are in the monomer regime, while unligated bradykinin B2 receptors on PC-12 cells are in the oligomer regime. In these examples, then, our (MC) model offers explanations for apparently contradictory data on receptor localization from several GPCR systems.

Next, we address the ability of our combined (MC+ODE) model to explain signaling data. Both signal amplification and attenuation have been reported as the effect of lipid rafts on different GPCR signaling systems. This is consistent with our combined (MC+ODE) model results for the influence of lipid raft coverage on the level of G-protein response (Supplementary Figure S4). For example, disruption of cell membrane lipid rafts attenuates the δ-opioid receptor-mediated signaling in brain neuronal cells, while enhances it in non-neuronal CHO cells [25]. Neurons in the brain have been demonstrated to be devoid of caveolae, but CHO cell line is a caveolae-rich cell line [24], [25], [62]. Non-caveolae rafts with their small size and cell surface coverage amplify G-protein signaling in neuronal cells, while caveolae with their relatively larger size and membrane coverage may attenuate it in CHO cells.

Recently, a FRET microscopy technique was used to reveal that functional neurokinin 1 receptors expressed in HEK293 cells are monomeric, concentrate in microdomains representing only 0.8–2.5% of the total cell surface area and do not dimerize upon agonist binding [30]. These observations are consistent with results of our MC model showing receptors in the monomer regime reside in lipid rafts. Moreover, our modeling indicates that receptor localization within G-protein enriched lipid rafts with small coverage (∼2%) leads to signal amplification, consistent with experimental data on neurokinin 1 receptor signaling in HEK293 cells [63].

Maximal effect (E_max_) and half maximal effective concentration (EC_50_) are commonly measured to compare the signaling efficacies of different ligands as well as potency of the ligands under different conditions. We calculated and compared E_max_ and EC50 for our model in the presence and absence of lipid rafts (Figure 6C). Both maximal effect and ligand potency increase (over the case of no lipid rafts) when lipid rafts at 2% coverage amplify the G-protein response. On the other hand, when lipid rafts at 30% coverage are compared to the case of no lipid rafts, although maximal effect decreases, EC50 is not significantly affected. In agreement with Figure 6C, disruption of lipid rafts (via cholesterol depletion) in systems with small (e.g. 2%) raft coverage has been observed to be accompanied by a decrease in both maximal effect (E_max_) and potency of the agonist [24], [63]. Further, cholesterol depletion has been shown to increase the maximal effect without significantly changing ligand potency when lipid raft disruption increases the level of G-protein response that occurs (based on our model) at a high (e.g. 30%) raft coverage [24], [57], [64].

### Modeling results can be tested via particular experimental protocols on GPCR signaling systems

Further experiments are required to rigorously test our model. Bioluminescence resonance energy transfer (BRET) and fluorescence resonance energy transfer (FRET) techniques have been used to provide quantitative information on either dimerization status or lateral distribution of receptors (e.g. lipid raft partitioning) in living cells or lipid vesicles [30], [65]–[67], but the correlation between the two has not yet been studied. Similar experiments that simultaneously examine both dimerization status and receptor distribution following dimerization-inducing/inhibiting treatments (e.g. ligand addition) in multiple GPCR systems are needed and could be compared with results of the MC model (Figures 3 and 7).

**Figure 8:**
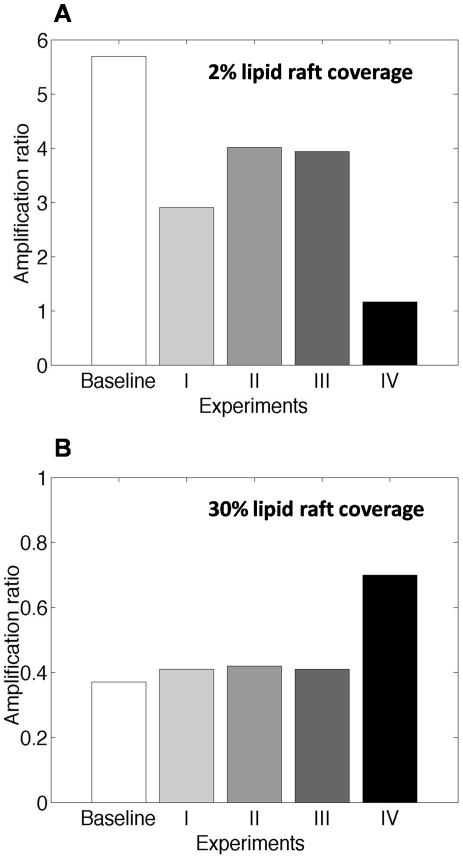
Simulation results for proposed experimental protocols for (A) amplification of G-protein signaling in the presence of lipid rafts with 2% coverage, and (B) attenuation of G-protein signaling in the presence of lipid rafts with 30% coverage. Baseline experiments are performed using *k_hyd_* = 10 s^−1^, *R_tot_* = 50,000 #/cell and [*L*] = 0.1*K_d_*. The effects of a change in a single parameter are shown by experiments I-III (experiment I∶*k_hyd_* = 1 s^−1^, experiment II∶*R_tot_* = 250,000 #/cell and experiment III∶[*L*] = *K_d_*). The effect of a simultaneous change in all three parameters is shown by experiment IV (*k_hyd_* = 1 s^−1^, *R_tot_* = 250,000 #/cell and [*L*] = *K_d_*). The greatest enrichment ratio for ligand-bound receptors predicted by MC model was used in each simulation. Other parameter values are as listed in Table 1.

In addition, our sensitivity analysis findings (Table 3) can be used to describe a paradigm to design experiments for testing our G-protein signaling model. The amount of lipid raft coverage, total number of cell surface receptors, GTP hydrolysis rate constant and ligand concentration were shown to affect the amplification ratio. Simulation results for a few experimental protocols based on these findings are described in Figure 8. As noted earlier, distinct results are expected for experiments on membranes with small and great lipid raft coverage. For example, when lipid rafts amplify G-protein signaling (i.e. lipid rafts cover ∼2% of the cell membrane), increasing *k_hyd_* (via RGS overexpression) and decreasing *R_tot_* (via receptor blockage) intensify signal amplification, while decreasing *k_hyd_* (via RGS inhibition) and increasing *R_tot_* (via receptor overexpression) are expected to decrease the amplification ratio. On the other hand, when the presence of lipid rafts leads to signal attenuation (i.e. lipid rafts cover 10–30% of the cell membrane), opposite effects are expected for similar variations in *k_hyd_* and *R_tot_*. As a result, both RGS inhibition and receptor overexpression are expected to neutralize the effect of lipid rafts on the level of response and thus diminish the influence of lipid raft disruption on G-protein signaling.

### Conclusion

We developed a kinetic model that quantitatively describes the effects of receptor dimerization and low diffusivity regions (lipid rafts) on GPCR organization and signaling. Although no direct experimental evidence yet exists for specific testing of results, our modeling demonstrates how ligands with particular dimerization-inducing or inhibiting characteristics may alter GPCR organization on the cell surface and in turn affect the level of G-protein activation. Depending on the unligated and ligated receptor dimerization and monomerization rate constants, ligand binding may quickly move receptors into or out of lipid rafts. Such re-organization of receptors may then enhance or diminish the GPCR-mediated response. Receptor phosphorylation can also be affected by the organization of GPCRs on the membrane as well (see Supplementary Text S2 and Figure S5). Thus receptor dimerization and lipid rafts may work together to provide a flexible platform for controlling both the extent and dynamics of GPCR signaling. A potentially powerful option for drug design for GPCR-associated diseases would be to tailor ligands to control receptor dimerization on the cell membrane in order to regulate G-protein signaling.

Our theoretical framework must be further validated in the context of experimental studies such as described in the text and Figure 8. However, our model already allows us to understand and connect individual observations in the literature on the role of receptor dimerization and lipid rafts in G-protein signaling. For example, we can provide explanations for experimental observations, including how various ligands differently re-organize δ-opioid receptors on the cell membrane [24], how dimerization-inducing ligands have distinct effects on localization of β2-adrenergic receptors and bradykinin B2 receptors relative to lipid rafts [12], and how lipid raft disruption amplifies G-protein signaling in a cell type but attenuates it in another type [24], [25].

Finally, we anticipate that other factors, including receptor hetero-dimerization, preferential interactions of GPCRs with particular membrane lipids, lipid raft dynamics and actin cytoskeleton re-arrangements, receptor cross-talk and G-protein independent pathways such as β-arrestin binding to receptors further increase the possible range of outcomes of this signaling system [52], [68]–[73].

## Supporting Information

Text S1 Monte Carlo simulation procedure
(0.40 MB DOC)

Text S2 Receptor phosphorylation can be regulated by lipid rafts.
(0.04 MB DOC)

Figure S1 Formation of oligomers via diffusion-limited partner switching.
(0.22 MB TIF)

Figure S2 Variation of average receptor-receptor interaction time with *k_mono_* and *k_dimer_*. MC Simulations were run with receptor density of 18% and membrane diffusion coefficient of 10^-9^ cm^2^/s. Dimensionless average interaction time is indicated by color.
(2.06 MB TIF)

Figure S3 Predicted variation of enrichment ratio with *k_mono_* and *k_dimer_* for different values of diffusion coefficient in lipid raft and non-raft regions of the cell membrane. Diffusion coefficients in lipid raft and non-raft regions are respectively (A) 10^−11^ cm^2^/s and 10^−10^ cm^2^/s, and (B) 10^−11^ cm^2^/s and 10^−9^ cm^2^/s. Simulations were run to equilibrium with receptor density of 18%. In this set of simulations, rafts make up 20% of the simulated membrane and raft diameter is 50 nm.
(2.70 MB TIF)

Figure S4 Scatter plots for the effect of relative G-protein density (*r*) on the model outcome, signal amplification ratio, at three levels of lipid raft coverage: (A) 2%, (B) 10%, and (C) 30%. Ranges for all other parameters are indicated in Table 1. The largest values of receptor dimerization-dependent enrichment ratio (found from MC simulations) were used.
(1.16 MB TIF)

Figure S5 Predicted effect of receptor localization within lipid rafts on the number of phosphorylated receptors in the membrane. Results are shown for two different values of receptor enrichment ratio (2.5 and 4.5 for low and high level of enrichment respectively) based on MC simulation results. Receptor clustering in lipid rafts enhances their G-protein dependent phosphorylation. *k_int_* for the fast and slow receptor internalization was assumed to be 10^−1^ s^−1^ and 10^−3^ s^−1^ respectively. Relative G-protein density in lipid rafts, *r*, was assumed to be 0.8. Membrane diffusivities in the raft and non-raft regions and lipid raft size and coverage are the same as Figure 6A. Other parameter values are as listed in Table 1.
(0.72 MB TIF)
