# Supplementary material for: Lipid Raft-Mediated Regulation of G-Protein Coupled Receptor Signaling by Ligands which Influence Receptor Dimerization: A Computational Study
Source: PLoS One. 2009 Aug 11;4(8):e6604. doi: 10.1371/journal.pone.0006604 (PMC2719103; doi:10.1371/journal.pone.0006604)
Supplement: Text S1 — Monte Carlo simulation procedure (0.40 MB DOC) [file pone.0006604.s001.doc]

**Monte Carlo (MC) simulation procedure**

The two-dimensional cell membrane surface was modeled as a 700 by 700 triangular lattice with periodic boundary conditions and a lattice spacing of 0.5 nm. Low diffusivity regions with uniform distribution and defined surface area (2-30% of the cell membrane) were designated as raft regions on the lattice. Simulations were initiated by placing receptor molecules (hexagons with a diameter of 5 nm, the approximate diameter of a single GPCR) randomly on the lattice. Placement on all lattice sites, whether in raft or non-raft regions, was equally likely. No two molecules were allowed to occupy the same lattice site. Thus, if the selected site was occupied, a new random lattice site was selected. Simulations were then run by random choosing of a single receptor and probability-based selection of an event (movement, dimerization, monomerization, ligand binding or unbinding) to occur at each time step. Possible events are limited to movement, dimerization and monomerization in the absence of ligand. The time step of each simulation was calculated from the fastest event in the system, either a reaction event or a move event controlled by diffusivity, and total number of receptors. Thus, the inverse of the rate constant for the fastest event, divided by total number of receptors, was used as the MC time step. The rate constant for move event was calculated as *k_move_* = 6*D*/*l*^2^; where *l* is the triangular lattice spacing and *D* is the diffusion coefficient. To ensure that the model results are independent of MC time step, simulations were run with time steps smaller than the MC time step and results were shown to be indistinguishable. Simulations were written in C++ and were run on a Mac Pro with 2 × 2.66 GHz Dual-Core Intel Xeon processors.

During the simulation, receptors were randomly picked at each time step to react (dimerization, monomerization, ligand binding or unbinding) and move based on probability of these events. The probability of dimerization (and monomerization) was the same for all ligand-bound receptors but different from unligated ones. The probability of movement was determined by the location of receptor (raft or non-raft region). Details of the simulation procedure during a single time step of simulation are described below.


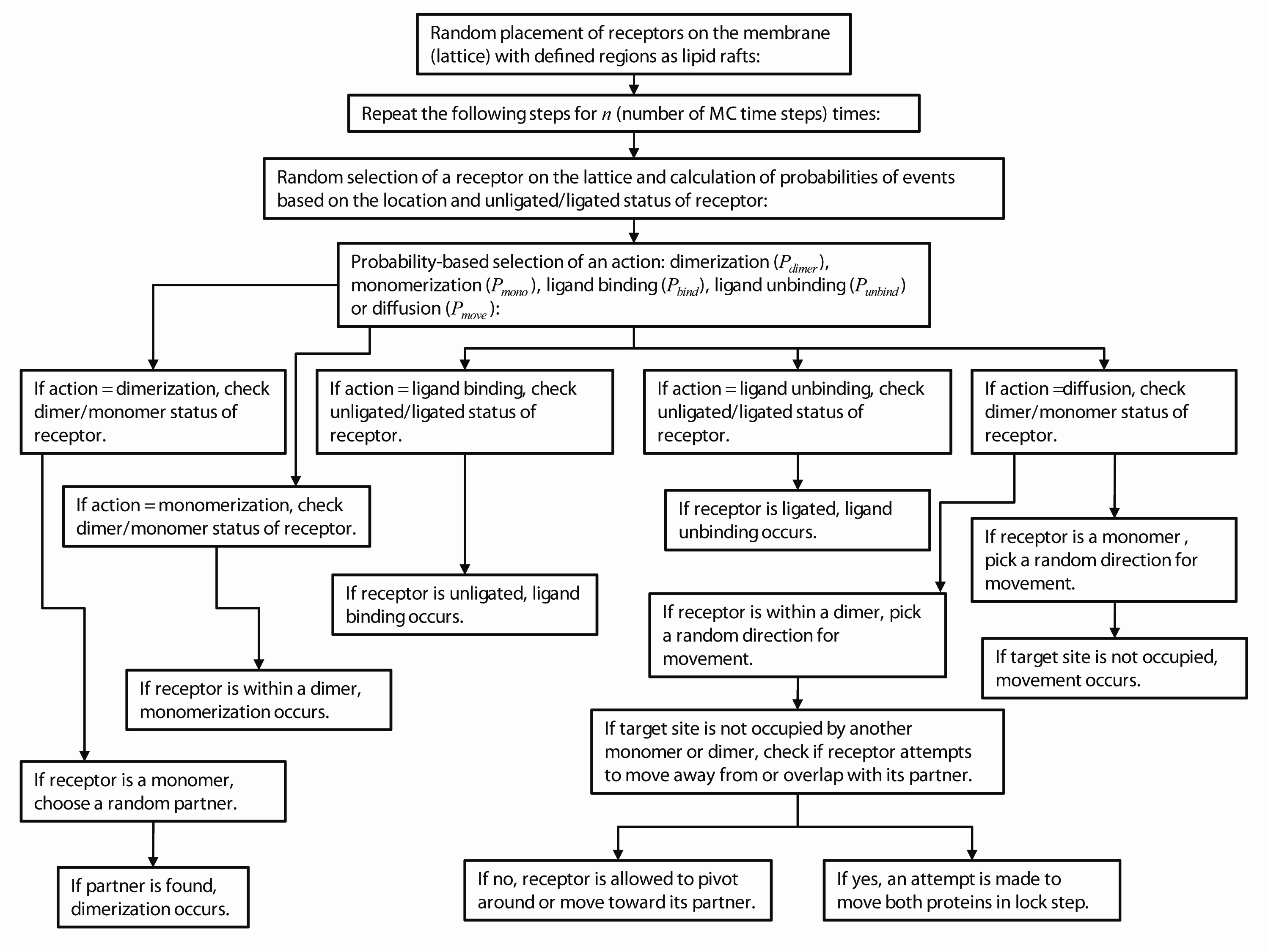


With a discrete-time model like the one used here, the dimerization and monomerization reactions can be modeled as Poisson processes. As a result, the probabilities of dimerization and monomerization can be derived from the reaction rate constants *k_dimer_* and *k_mono_* using a Poisson distribution. With sufficiently small time-steps (Δ*t*), this relationship simplifies such that the probability of a reaction is approximately proportional to the reaction rate constant as shown below ^[1]^.

$$P_{mono}=1-e^{-k_{mono}\Delta t}\approx k_{mono}\Delta t$$

$$P_{\dim er}=1-e^{-k_{\dim er}\Delta t}\approx k_{\dim er}\Delta t$$

Similarly, the probabilities of ligand binding to receptors and ligand unbinding from receptors can be calculated using the following equations:

$$P_{bind}=1-e^{-k_{f}[L]\Delta t}\approx k_{f}[L]\Delta t$$

$$P_{unbind}=1-e^{-k_{r}\Delta t}\approx k_{r}\Delta t$$

where, [*L*] is the ligand concentration and *k_f_* and *k_r_* are ligand/receptor association and dissociation rate constants, respectively.

The probability of a diffusion event, *P_move_*, was calculated using the translational diffusion coefficient *D_t_* of the membrane for GPCRs. For a single particle exhibiting Brownian diffusion on a triangular lattice, the probability of a particle moving at least one lattice spacing, *l*, in one iteration time step, Δ*t*, can be expressed as:

$$P_{move}=1-\exp(\frac{-6D_{t}\Delta t}{l^{2}})\approx\frac{6D_{t}\Delta t}{l^{2}}$$

Thus, at small time step values, the probability of a move is nearly proportional to the diffusion coefficient of the cell membrane which was assumed to be smaller for the lipid rafts compared with the non-raft region.

References

1.     Rowley RL. (1994) Statistical mechanics for thermophysical property calculations. Englewood Cliffs, NJ: Prentice-Hall.
