# Supplementary material for: Lipid Raft-Mediated Regulation of G-Protein Coupled Receptor Signaling by Ligands which Influence Receptor Dimerization: A Computational Study
Source: PLoS One. 2009 Aug 11;4(8):e6604. doi: 10.1371/journal.pone.0006604 (PMC2719103; doi:10.1371/journal.pone.0006604)
Supplement: Text S2 — Receptor phosphorylation can be regulated by lipid rafts. (0.04 MB DOC) [file pone.0006604.s002.doc]

**Receptor phosphorylation can be regulated by lipid rafts**

Using the ODE model (Figure 2B) we also asked whether dimerization-mediated receptor clustering in lipid rafts affects receptor phosphorylation. Receptor clustering into lipid rafts with a high concentration of G-protein relative to the non-raft region (*r* = 0.8) may enhance their diffusion-limited phosphorylation by kinases (GRKs) recruited by βγ-subunits of G-proteins. However, the level of enhancement in the number of phosphorylated receptors in the cell membrane depends on the rate of receptor internalization (Supplementary Figure S5). Recent experiments have shown that dimerization of the thyrotropin-releasing hormone (TRH) receptor potentiates hormone-dependent GRK-mediated receptor phosphorylation ^[1]^. Although disruption of the plasma membrane integrity by cholesterol depletion has been reported to impair the effectiveness of TRH signaling via G-proteins ^[2]^, no data are available on how dimerization of TRH receptors influences their organization on the membrane. Our combined model of receptor dimerization and G-protein signaling can connect these experimental observations and predicts the increase in the level of receptor phosphorylation due to dimerization-mediated enrichment of receptors in lipid rafts.

**References**

1.     Song GJ, Jones BW, Hinkle PM. (2007) Dimerization of the thyrotropin-releasing hormone receptor potentiates hormone-dependent receptor phosphorylation. Proc Natl Acad Sci U S A 104(46): 18303-18308.

2.     Ostasov P, Bourova L, Hejnova L, Novotny J, Svoboda P. (2007) Disruption of the plasma membrane integrity by cholesterol depletion impairs effectiveness of TRH receptor-mediated signal transduction via G(q)/G(11)alpha proteins. J Recept Signal Transduct Res 27(5-6): 335-352.
