# Supplementary figures and images for: Lipid Raft-Mediated Regulation of G-Protein Coupled Receptor Signaling by Ligands which Influence Receptor Dimerization: A Computational Study
Source: PLoS One. 2009 Aug 11;4(8):e6604. doi: 10.1371/journal.pone.0006604 (PMC2719103; doi:10.1371/journal.pone.0006604)

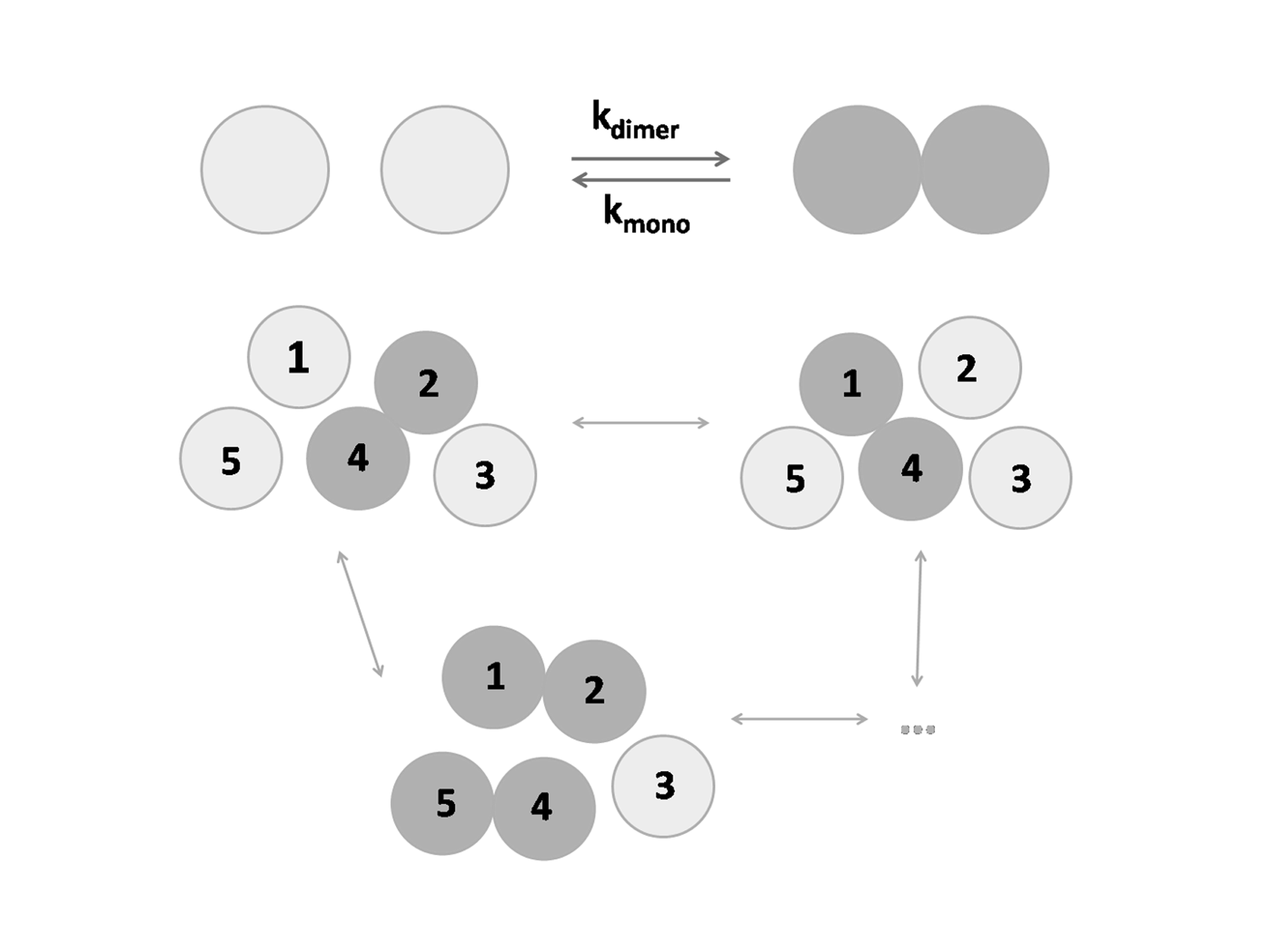

Supplement: Figure S1 — Formation of oligomers via diffusion-limited partner switching. (0.22 MB TIF) [file pone.0006604.s003.tif]

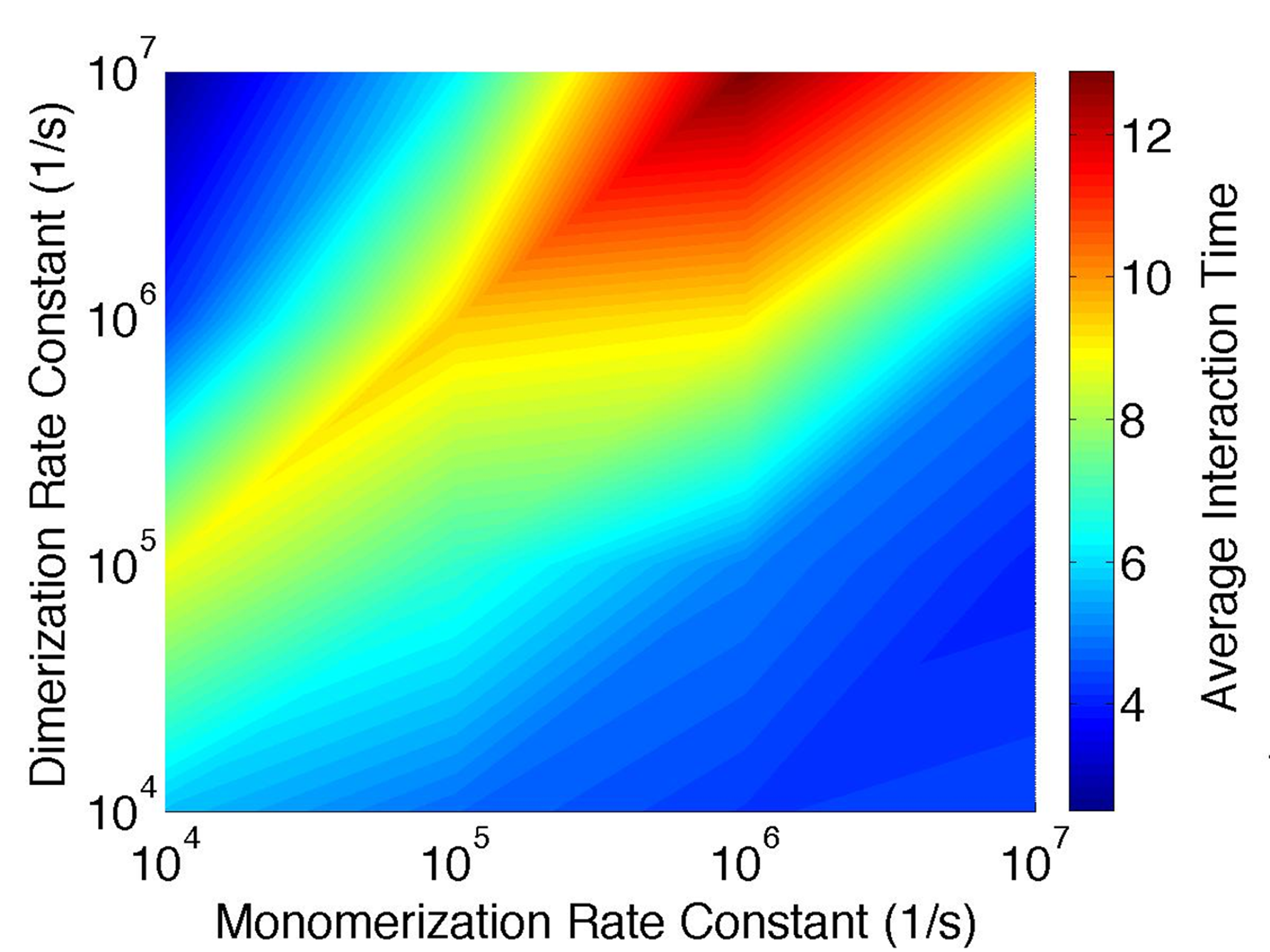

Supplement: Figure S2 — Variation of average receptor-receptor interaction time with kmono and kdimer. MC Simulations were run with receptor density of 18% and membrane diffusion coefficient of 10-9 cm2/s. Dimensionless average interaction time is indicated by color. (2.06 MB TIF) [file pone.0006604.s004.tif]

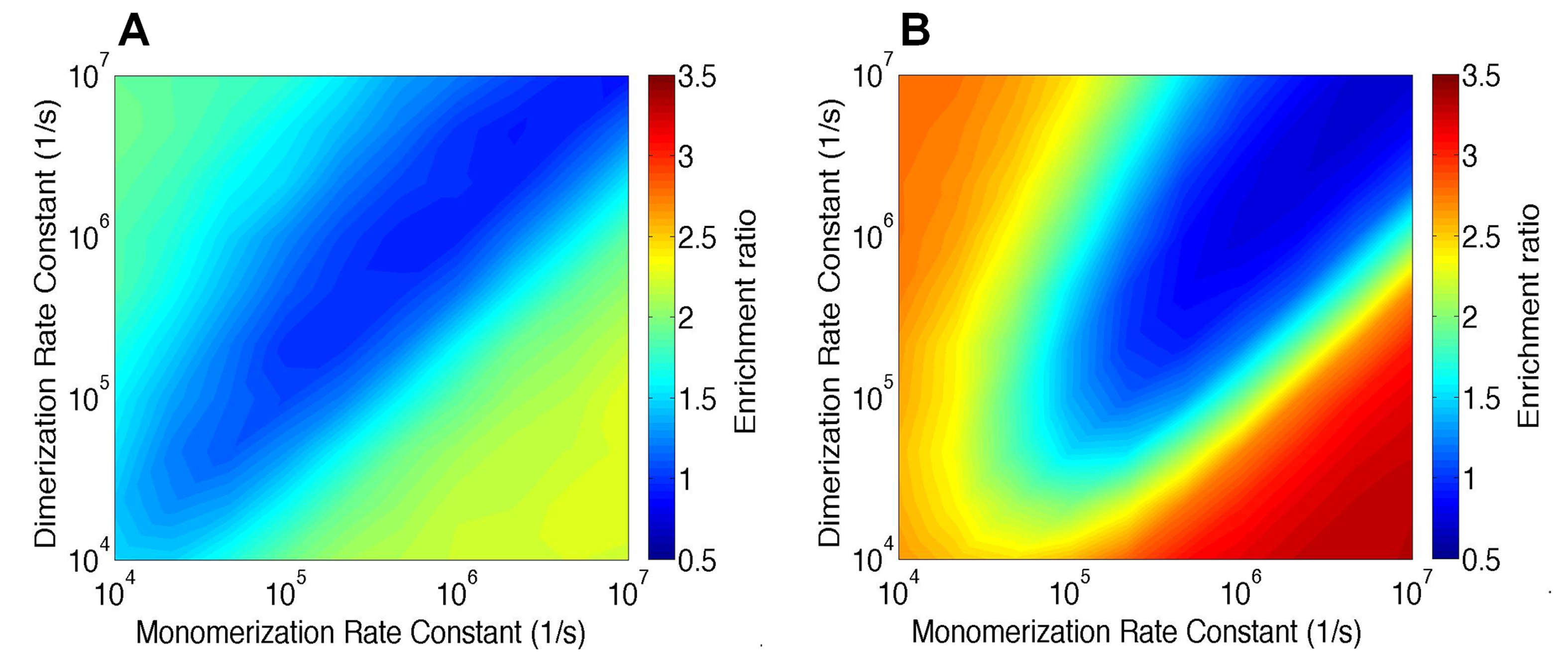

Supplement: Figure S3 — Predicted variation of enrichment ratio with kmono and kdimer for different values of diffusion coefficient in lipid raft and non-raft regions of the cell membrane. Diffusion coefficients in lipid raft and non-raft regions are respectively (A) 10−11 cm2/s and 10−10 cm2/s, and (B) 10−11 cm2/s and 10−9 cm2/s. Simulations were run to equilibrium with receptor density of 18%. In this set of simulations, rafts make up 20% of the simulated membrane and raft diameter is 50 nm. (2.70 MB TIF) [file pone.0006604.s005.tif]

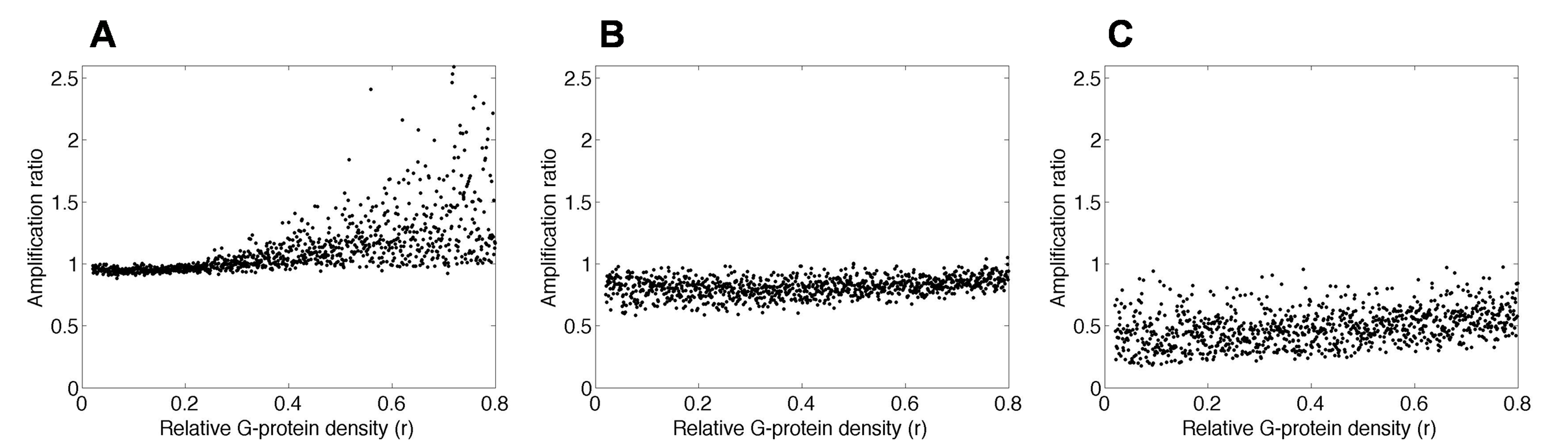

Supplement: Figure S4 — Scatter plots for the effect of relative G-protein density (r) on the model outcome, signal amplification ratio, at three levels of lipid raft coverage: (A) 2%, (B) 10%, and (C) 30%. Ranges for all other parameters are indicated in Table 1. The largest values of receptor dimerization-dependent enrichment ratio (found from MC simulations) were used. (1.16 MB TIF) [file pone.0006604.s006.tif]

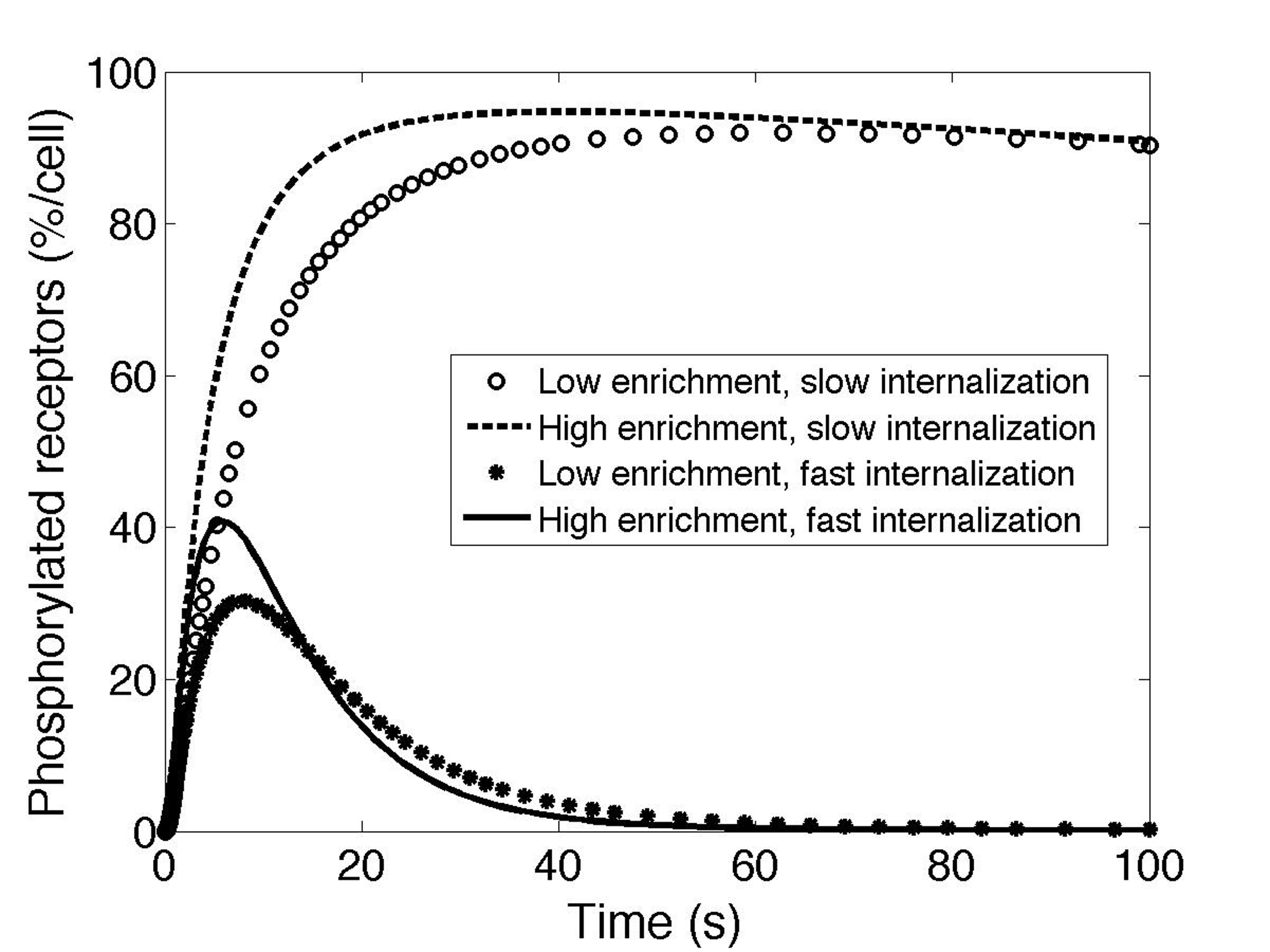

Supplement: Figure S5 — Predicted effect of receptor localization within lipid rafts on the number of phosphorylated receptors in the membrane. Results are shown for two different values of receptor enrichment ratio (2.5 and 4.5 for low and high level of enrichment respectively) based on MC simulation results. Receptor clustering in lipid rafts enhances their G-protein dependent phosphorylation. kint for the fast and slow receptor internalization was assumed to be 10−1 s−1 and 10−3 s−1 respectively. Relative G-protein density in lipid rafts, r, was assumed to be 0.8. Membrane diffusivities in the raft and non-raft regions and lipid raft size and coverage are the same as Figure 6A. Other parameter values are as listed in Table 1. (0.72 MB TIF) [file pone.0006604.s007.tif]
